# Supplementary material for: Iterative improvement in the automatic modular design of robot swarms
Source: PeerJ Comput Sci. 2020 Dec 7;6:e322. doi: 10.7717/peerj-cs.322 (PMC7924708; doi:10.7717/peerj-cs.322)
Supplement: Supplemental Information 3 [file peerj-cs-06-322-s003.zip › argos3/doc/api/standalone/a00530_source.html]

ARGoS: plugins/robots/generic/simulator/leds\_default\_actuator.cpp Source File


- Main Page
- Related Pages
- Namespaces
- Classes
- Files

- File List
- File Members

# plugins/robots/generic/simulator/leds\_default\_actuator.cpp

Go to the documentation of this file.

```
00001 
00007 #include "leds_default_actuator.h"
00008 #include <argos3/core/simulator/simulator.h>
00009 #include <argos3/core/utility/logging/argos_log.h>
00010 #include <argos3/plugins/simulator/media/led_medium.h>
00011 
00012 namespace argos {
00013 
00014    /****************************************/
00015    /****************************************/
00016 
00017    CLEDsDefaultActuator::CLEDsDefaultActuator() :
00018       m_pcLEDEquippedEntity(NULL) {}
00019 
00020    /****************************************/
00021    /****************************************/
00022 
00023    void CLEDsDefaultActuator::SetRobot(CComposableEntity& c_entity) {
00024       m_pcLEDEquippedEntity = &(c_entity.GetComponent<CLEDEquippedEntity>("leds"));
00025       m_tSettings.resize(m_pcLEDEquippedEntity->GetLEDs().size());
00026    }
00027 
00028    /****************************************/
00029    /****************************************/
00030 
00031    void CLEDsDefaultActuator::Init(TConfigurationNode& t_tree) {
00032       try {
00033          CCI_LEDsActuator::Init(t_tree);
00034          std::string strMedium;
00035          GetNodeAttribute(t_tree, "medium", strMedium);
00036          m_pcLEDMedium = &CSimulator::GetInstance().GetMedium<CLEDMedium>(strMedium);
00037          m_pcLEDEquippedEntity->AddToMedium(*m_pcLEDMedium);
00038       }
00039       catch(CARGoSException& ex) {
00040          THROW_ARGOSEXCEPTION_NESTED("Error initializing the LEDs default actuator", ex);
00041       }
00042    }
00043 
00044    /****************************************/
00045    /****************************************/
00046 
00047    void CLEDsDefaultActuator::Update() {
00048       m_pcLEDEquippedEntity->SetAllLEDsColors(m_tSettings);
00049    }
00050 
00051    /****************************************/
00052    /****************************************/
00053 
00054    void CLEDsDefaultActuator::Reset() {
00055       SetAllColors(CColor::BLACK);
00056    }
00057 
00058    /****************************************/
00059    /****************************************/
00060 
00061    void CLEDsDefaultActuator::Destroy() {
00062       m_pcLEDEquippedEntity->RemoveFromMedium();
00063    }
00064 
00065    /****************************************/
00066    /****************************************/
00067 
00068 }
00069 
00070 REGISTER_ACTUATOR(CLEDsDefaultActuator,
00071                   "leds", "default",
00072                   "Carlo Pinciroli [ilpincy@gmail.com]",
00073                   "1.0",
00074                   "The LEDs actuator.",
00075                   "This actuator controls a group of LEDs. For a complete description of its\n"
00076                   "usage, refer to the ci_leds_actuator.h file.\n\n"
00077                   "REQUIRED XML CONFIGURATION\n\n"
00078                   "  <controllers>\n"
00079                   "    ...\n"
00080                   "    <my_controller ...>\n"
00081                   "      ...\n"
00082                   "      <actuators>\n"
00083                   "        ...\n"
00084                   "        <leds implementation=\"default\"\n"
00085                   "              medium=\"leds\" />\n"
00086                   "        ...\n"
00087                   "      </actuators>\n"
00088                   "      ...\n"
00089                   "    </my_controller>\n"
00090                   "    ...\n"
00091                   "  </controllers>\n\n"
00092                   "The 'medium' attribute sets the id of the LED medium declared in the <media>\n"
00093                   "XML section.\n\n"
00094                   "OPTIONAL XML CONFIGURATION\n\n"
00095                   "None.\n",
00096                   "Usable"
00097    );
00098
```

---

Generated on 10 Jul 2018 for ARGoS by 
 1.6.1
